# Starvation Stress Causes Body Color Change and Pigment Degradation in *Acyrthosiphon pisum*

**DOI:** 10.3389/fphys.2019.00197

**Published:** 2019-03-05

**Authors:** Xing-Xing Wang, Zhan-Sheng Chen, Zhu-Jun Feng, Jing-Yun Zhu, Yi Zhang, Tong-Xian Liu

**Affiliations:** ^1^Key Laboratory of Integrated Pest Management on Crops in Northwestern Loess Plateau, Ministry of Agriculture, College of Plant Protection, Northwest A&F University, Yangling, China; ^2^College of Horiculture, Northwest A&F University, Yangling, China

**Keywords:** pea aphid, color fading, starvation, adaptation, pigment degradation

## Abstract

The pea aphid, *Acyrthosiphon pisum* (Harris), shows body color shifting from red to pale under starvation in laboratory conditions. These body color changes reflect aphid’s adaptation to environmental stress. To understand the color-shifting patterns, the underlying mechanism and its biological or ecological functions, we measured the process of *A. pisum*’s body color shifting patterns using a digital imagery and analysis system; we conducted a series of biochemical experiments to determine the mechanism that causes color change and performed biochemical and molecular analyses of the energy reserves during the color shifting process. We found that the red morph of *A. pisum* could shift their body color to pale red, when starved; this change occurred rapidly at a certain stress threshold. Once *A. pisum* initiated the process, the shifting could not be stopped or reversed even after food was re-introduced. We also discovered that the orange-red pigments may be responsible for the color shift and that the shift might be caused by the degradation of these pigments. The carbohydrate and lipid content correlated to the fading of color in red *A. pisum*. A comparative analysis revealed that these reddish pigments might be used as backup energy. The fading of color reflects a reorganization of the energy reserves under nutritional stress in *A. pisum*; surprisingly, aphids with different body colors exhibit diverse strategies for storage and consumption of energy reserves.

## Introduction

Many animals, including insects, show variations in their body colors. This coloration could be used in making mating choices, sending warning signals, receiving external radiant energy for body temperature regulation, and many other functions (Huey and Kingsolver, 1989; Merilaita and Tullberg, 2005; Forsman et al., 2008). Aphids that show polyphenism in their sexual generation, wing formation, and body colors are flexible under varied environmental conditions. The pea aphid, *Acyrthosiphon pisum* (Harris), generally contains two strains based on body color, red and green. Ecological studies showed that red aphids on green plants are easily detected by predators, while green aphids can be easily parasitized by parasitoids (Losey et al., 1997; Libbrecht et al., 2007). The green peach aphid, *Myzus persicae* (Sulzer), is known to display a connection between body color and esterase differences (Takada, 1979); the red strain of *A. pisum* might use its pigments (carotenoid pigments) for light-induced electron transfer and ATP synthesis (Valmalette et al., 2012).

Body color in aphid species is affected by many physical conditions. Studies on the English grain aphid, *Sitobion avenae* (Fabricius), showed that the aphid changes its body color from green to pink or brown with changes in light intensity (Alkhedir et al., 2010). Also, the red *A. pisum* can shift color to green in low temperatures within a few generations (Valmalette et al., 2012). Other studies showed that the body colors of *M. persicae* and *S. avenae* can also be affected by temperature (Matsumoto and Tsuji, 1979; Takada, 1981).

Biological conditions can similarly modify aphid body color. For instance, different color-esterase morphs in *M. persicae* might be determined by their hosts (Takada, 1979). In addition, an endosymbiont *Rickettsiella* sp. was reported to be responsible for the change of *A. pisum* body color from red to green over one generation (Tsuchida et al., 2010).

The insect’s body color is basically determined by several kinds of chemical pigments. Aphid pigments that make them colorful comprise of two categories. The first category includes aphins, which specifically exist in the hemolymph of aphids (Bowie et al., 1966) and have multiple molecular structures with different colors, such as red or green, and constitute the base color for aphids (Bowie et al., 1966; Horikawa et al., 2004; Horikawa et al., 2006; Alkhedir et al., 2010). The second category includes carotenoid pigments, such as β-carotene, lycopene, and torulene, which are present in lower quantities than aphins, and are synthesized within aphids, and thus, can strongly affect body color (Andrewes et al., 1971; Jenkins et al., 1999; Moran and Jarvik, 2010). The pea aphids show a relatively stable red-green color polymorphism. They can make their own carotenoids based on the genes laterally transferred from fungi. Studies have shown red individuals to have a single carotenoid desaturase enzyme that is absent from green individuals (Moran and Jarvik, 2010). Laboratory crosses between the two strains and a Mendelian genetic analysis revealed that color polymorphism in the pea aphids is determined by a single bi-allelic locus named *colorama* (Caillaud and Losey, 2010). Previous studies have shown that the host plant might also affect the aphid’s color. For instance, Losey and Eubanks (2000) found that the color morphs of pea aphids were not significantly different from those collected from forage crops and various vegetables. However, the frequency of occurrence of these color morphs was different on the three hosts: *Pisum sativum* (pea), *Medicago sativa* (alfalfa), and *Trifolium pratense* (red clover; Simon et al., 2003). Although the two morphs are stable, the red individuals can turn into green at low temperatures (Valmalette et al., 2012) or following an infection of endosymbionts (Tsuchida et al., 2010).

Another color-changing phenomenon exists in the red *A. pisum*, which is the shifting of body color from red (pink) to pale red; this change is reversible, even though the change is directly associated with the change in carotenoids (Valmalette et al., 2012; Tabadkani et al., 2013). The color shifting in red *A. pisum* could be triggered by low-quality diets and can be reversed, when nutrition habits change from a low-quality to a high-quality diet. The pea aphids with pale body color have significantly lower wet and dry weights, which may be attributable to a loss of lipids and soluble carbohydrates, as well as faster movements; in addition, this color reversion enables the aphids to quickly respond to deprived host plants and restore their original status, when they find appropriate host plants (Tabadkani et al., 2013). The shift in color in this process may be a type of stress symptom or may possibly play a role in response mechanisms to environmental threats.

In red *A. pisum*, body color represents the aphid’s response to nutritional stress; scant information is available on the mechanism behind the shift from red to pale red. Previous studies simply divided the aphids into red (pink) or pale red categories. Furthermore, the ecological and biological functions of these changeable pigments are also unclear. In our study, we monitored the color shift process in *A. pisum* under starvation and revealed the details of this shift. In addition, we extracted the red pigments from aphids at every color-shifting stage for further analysis. Considering the connection between color changes and nutritional decline, the content changes of energy reserves (soluble carbohydrate, glycogen, lipid, and protein) and its correlation with body color at different color-shifting stages were also measured. We also conducted an analysis of the energy reserves to evaluate the potential function of reddish pigments by comparing the red and green strains of *A. pisum* under starvation.

## Materials and Methods

### Experimental Insects and Plants

A red strain of the pea aphid, *A. pisum*, was collected from Lanzhou, Gansu Province, northwestern China, while a green strain was obtained from our laboratory colony in Yangling, Shaanxi, China. Both strains were cultured on broad bean (*Vicia faba* L., var. “Jinnong”) as a substrate under long-day conditions (16L: 8D; 20 ± 1°C) for more than 30 generations at the Key Laboratory of Applied Entomology, Northwest A&F University, Yangling, Shaanxi, China. All aphids were reared at a low density (less than 30 individuals per plant) for more than three generations before they were subjected to the experiments below. As compared to nymphs, *A. pisum* adults show additional behaviors, such as nymph-production or dispersal (winged), which make their energy consumption more complicated; therefore, wingless fourth-instar nymphs (12 h after molting) were picked for experiments.

### Aphid Color Collection

#### Color Collection From Color-Shifted Aphids

In order to get color data, we used digital camera observing images for further analysis (Joiner, 2004; Murakami et al., 2005; Lebourgeois et al., 2008). Nutrition stress is one important stimulation for shifting of body color in our experience, but it is difficult to sustain a stable nutrition stress under laboratory conditions for analysis. We picked starvation as a stable and controllable nutrition stress in our treatments, which is also common in wild conditions. Starvation treatment is stable enough for laboratory experimentation and is easy to control. Fourth-instar nymphs (12 h after molting) of the wingless red *A. pisum* were placed in a transparent plastic dish (90 mm in diameter) and a 24-well tissue culture plate (transparent, plastic, one individual per well) depending on the experiments. The aphids were starved for 24 h to stimulate shift in the body color. All devices were placed under 24-h light conditions (20 ± 1°C). A cold light source (KL 1500 LCD, Zeiss, Germany, temperature of 3200 K) was used for lighting. Images were taken using a digital camera (Canon^®^ EOS 5D Mark III, Canon, Tokyo, Japan; Lens: Canon^®^ Macro lens EF 100 mm 1:2.8 L IS USM, Canon, Japan). The camera parameters were set as instructed in the manufacturer’s manual (shutter speed: 1/250; aperture: F4.0; ISO: 320; picture style: faithful 0,0,0,0; white balance: color temp, 3200 K, AF mode: manual focus; metering mode: center-weighted average), and the images were recorded in RAW (.CR2); and the 24-Patch ColorChecker chart (Mennon, China) with standard colors on was used to correct images before experiments (Supplementary Figure S1A). The images were read and modified by Adobe Camera Raw and corrected by the 24-Patch ColorChecker chart in Adobe DNG Profile Editor, and the values of RGB channels (RGB for red, green and blue, respectively, as below) were read and recorded by Adobe Photoshop CS6 and Microsoft Excel 2013 (Schwarz et al., 1987; Zhang et al., 2003).

##### Color collection during aphid color shifting

To analyze the body color shifting pattern, 50 12-h-old wingless fourth instar nymphs of the red *A. pisum* (well-cultured and in low density) were used in each color shifting test. The new-molting nymphs were selected and reared in host plants for 12 h, and then collected and placed in a transparent plastic dish (90 mm in diameter; 10 individuals per dish) for starvation treatment. The color-shifting images of the aphids were captured at hourly intervals for 24 h. The values of RGB channels were read by Adobe Photoshop CS6 software.

##### Body color reversibility test

To understand if the color shifting could be reversed or not, 58 12-h-old wingless fourth instar nymphs of the red *A. pisum* (well-cultured and in low density) were individually picked into two 24-well tissue culture plates (transparent plastic; 18 mm in diameter of each well; one individual per well) for each starvation treatment. Aphid images were captured at an early stage (12 h after treatment began) and late stage (24 h after treatment began). To determine if the color of the aphids can be reversed after they are re-introduced to food, images of the aphids were collected in a condition of starvation, and then they were re-transferred onto *V. faba* leaf disks (with 1% agar) in two 24-well tissue culture plates and reared on the leaf within them. Images of the aphids were then captured after 24 h of being reared on the leaves to analyze changes in body color.

### Pigment Extraction and Analysis

For a deeper understanding of the composition of body pigments and the changes in the shifting process, a series of studies were performed.

#### Extraction of Pigments for Spectral Analysis

The protocol of extraction has been presented before in Tsuchida et al. (2010) and Valmalette et al. (2012) and it was modified before use. The 12-h-old wingless fourth-instar nymphs of the red *A. pisum* strain were starved for 16 h to stimulate red to pale red color shift. The pale individuals (whose body color had changed) were collected for extraction of pigments; the red individuals were collected from prepared aphids without starvation. Collected samples were quick-frozen by liquid nitrogen and freeze-dried for 36 h in a lyophilizer (MSA3.6P, Sartorius, Germany). The pigments were then extracted with chloroform and ethyl alcohol. Following this, 20 mg of each sample was transferred into a 1.5 mL microtube and homogenized by a micropestle in 1 mL of chloroform. All samples were kept in the dark for 24 h. The supernatant was filtered into a new 1.5-mL microtube using a 1-mL injector and a nylon syringe filter (this mixture of pigments was marked as “pigment mix”). The solution was then condensed in a speed vacuum concentrator (ScanSpeed 40, Scanvac, Denmark; 2000 × *g* 1 h, 40°C) until all red pigments separated out; 200 μL of ethyl alcohol was added to dissolve the solute by mixing for 2 h; the contents were then centrifuged at 20,000 × *g* for 15 min at 4°C, and the supernatant was transferred into a new tube. The ethyl alcohol-insoluble deposits were washed twice with 1 mL of ethyl alcohol, followed by addition of 50 μL of chloroform to dissolve the remainder (this pigment mixture recognized as “tomato red” was marked Pigment 1); the supernatant which was transferred to a new tube was condensed in a speed vacuum concentrator till all solutes separated out and then resolved using 200 μL of chloroform (this pigment mixture that was recognized as “orange-red” was marked Pigment 2; Supplementary Figure S1B).

#### Pigment Absorbance Patterns

The 12-h-old wingless fourth-instar nymphs were starved for 16 h to stimulate the red to pale red color shift. The aphids were then individually placed into transparent plastic dishes (90 mm in diameter; 20 individuals per dish). The aphids were collected at 1-h intervals for 3 h. The collected samples were used for pigment extraction. UV–Vis absorbance spectra of the pigment solution were then collected using Nanodrop 2000c spectrophotometer (Thermo Fisher Scientific Inc., United States).

#### Correlation of Pigment Absorbance and Three RGB Color Channels

The images of the wingless fourth-instar nymphs in different body colors were captured using a digital camera, and the aphids were then used for pigment extraction. The values for the RGB color channels and the corresponding absorbance at λ_max_ (483 nm) were used for correlation analysis for the pigment 2 mixture.

#### Thin-Layer Chromatography of Aphid Pigments

The crude mixture of pigment extracts was used for thin-layer chromatography (TLC). The protocol of TLC was modified from Tsuchida et al. (2010). Based on the values for the G channel (as recorded using a digital camera), the aphids were categorized to have two colors: the red (*G* value: around 160) and the pale red (*G* value: around 190). The aphids were quick-frozen in liquid nitrogen and freeze-dried for 36 h in a lyophilizer (MSA3.6P, Sartorius, Germany). They were extracted using a chloroform-ethyl alcohol (1:2) mixture. The total solution was kept in the dark for 24 h and centrifuged at 20,000 × *g* for 15 min at 4°C to remove deposition from the mixture. The clarified liquid was used for TLC.

### Red Pigment Extraction

The aphids were categorized according to the two body colors as described above; they were quick-frozen in liquid nitrogen and freeze-dried for 36 h in a lyophilizer (MSA3.6P, Sartorius, Germany). The samples were then used for extraction of the two red pigments (mixture of the pigments 1 and 2; Supplementary Figure S1B). Pigment mixtures from aphids with different body colors were then used for TLC.

### Thin-Layer Chromatography

The extraction solutions were subjected to phase TLC (pTLC) on pre-coated silica gel plates using chloroform-methanol (1:1) mixture as a solvent for all pigments and ethyl alcohol-water-ammonia-isopropyl alcohol-ethyl acetate (6:1:1:6:1) mixture for red pigments. The images of TLC were captured by a digital camera under visible and ultraviolet (295–365 nm) lights, which were then analyzed using the Adobe Photoshop CS6 software (Version 13.0 x64, Adobe, CA, United States).

### Energy Reserves Assay

Considering that nutrition stress is a key trigger for the fading of color, pigment degradation and conversion are probably related to nutritional physiology. Studies of energy reserves were performed.

#### Energy Reserves Assay

Twelve-hour-old fourth instar nymphs of the wingless red and green *A. pisum* were transferred into transparent plastic dishes (90 mm in diameter; 20 aphids per dish) for starvation treatment. The aphids were collected at hourly intervals for 24 h. The collected samples were then immediately used in energy reserve assays. The aphids were freeze-dried for 36 h in a lyophilizer (Heto PowerDry LL3000 Freeze Dryer, Thermo Fisher Scientific, United States); 2 mg of dried samples was weighed out using a high-precision electronic balance (MSA3.6P, Sartorius, Germany) at ambient temperature. The aphids were transferred into a 1.5-mL microtube, homogenized by a micropestle, and dissolved in 800 μL of solution buffer (100 mM KH_2_PO_4_, 1 mM DTT, 1 mM EDTA, pH 7.4) for further analysis. This experimental step had three replicates.

### Glycogen and Soluble Carbohydrate Assays

After all samples (2 mg of dried samples) were dissolved in the solution buffer as mentioned above, each tube with the aphid samples was centrifuged at 20,000 × *g* for 15 min at 4°C to remove deposition from the mixture. The supernatant was then transferred into a new tube for soluble carbohydrate assay; the deposits were washed twice using methanol for glycogen assay. Both glycogen and soluble carbohydrates were measured using the colorimetric method based on the enthrone reagent with glucose as the standard as described in Foray et al. (2012).

#### Protein Assay

After all samples (2 mg of dried samples) were dissolved in the solution buffer, each tube with the samples was centrifuged at 1800 × *g* for 15 min at 4°C. The protein content in these mixtures was measured using a modified Bradford protein assay kit (C503041-1000, Sangon Biotech, Shanghai, China). After preparation of the mixture, 2.5 μL of each supernatant was transferred into a 96-well microplate, together with 250 μL of Bradford reagent. Protein concentration was determined by a microplate reader (infinite M200, TECAN, Switzerland) at 595 nm.

#### Lipid Assay

After all samples (2 mg of dried samples) were dissolved in the solution buffer, 160 μL of NaOH (6 N) was added to hydrolyze the fat content in each tube; the tubes were water-bathed (75°C) for 3 h. The hydrolytic fatty acid content in the mixtures was measured using a non-esterified free fatty acids (NEFA) assay kit (A042, Nanjing Jiancheng Bioengineering Institute, Nanjing, China). The raw sample volume of each test was 0.2 mL. First, the prepared mixture was transferred into glass tubes and buffer B (0.5 mL) was added, followed by reagent C (1 mL with Cu^2+^; in blue color); after 4 mL of reagent A was added, samples were centrifuged at 3500 × *g* for 10 min (all these reagents are named by Nanjing Jiancheng Bioengineering Institute). Afterward, the supernatant was discarded, while 2 mL of clear liquid from the lower layer was transferred to a quartz cuvette for further analysis. Concentration of fatty acids was determined by NanoDrop^®^ 2000c (Thermo Fisher Scientific, Middletown, VA, United States) at 440 nm.

#### Color-Dependent Energy Reserves Assay (Red Strain)

New molt wingless aphids at the fourth-instar stage were collected and prepared for energy reserves assay. After 12 h of rearing, samples were transferred into a tissue culture plate (transparent, plastic, one individual per well) and starved for 12, 16, and 24 h; images of all aphids were then taken for color assay. Individuals from each treatment (12, 16, and 24 h) were divided into two groups (red and pale red) based on the values for the G channel (around 160 as red and 190 as pale red) observed in the images. Collected samples with three replications were then marked and used for energy reserve assays immediately. The protocols for this assay have been detailed in the previous section.

### Transcriptional Analyses

Glycogen was considered to play an important role in this process, and we have conducted a preliminary study of its downstream metabolism. The two aphid strains were starved as described above. Aphid samples were collected at hourly intervals for 24 h. Samples were quick-frozen using liquid nitrogen immediately after collection. Samples of RNA were extracted with RNAiso Plus (Takara, Japan), and cDNA was synthesized from them using a PrimeScript^TM^ RT reagent kit with gDNA Eraser (Takara, Japan). Quantitative real-time PCR (qRT-PCR) was performed with SYBR^®^ Premix Ex Taq^TM^ II (Takara, Japan) in an IQ-5 system (Bio-Rad, United States). Glycogen phosphorylase (EC 2.4.1.1) catalyzes the rate-limiting step in glycogenolysis in animals by releasing glucose-1-phosphate from the terminal alpha-1,4-glycosidic bond and breaks up glycogen into glucose subunits^1^. It is the key enzyme for glycogen metabolism. We selected it for evaluation of the Glycogen metabolism. *Ribosomal protein L7* (*Rpl7*) was selected as a reference gene from Nakabachi et al. (2005) and Guo et al. (2016). The primers were designed by Primer-BLAST available online at NCBI^2^.

Primer (*glycogen phosphorylase*, *GP*, ACYPI001125; *ribosomal protein L7*, *Rpl7*, ACYPI010200) sequences were as follows:

GP-f: GCTCAGAAAATAACCAACGG; GP-r: GTGTGTCTACTACTTTGCCA
Rpl7-f: GCGCGCCGAGGCTTAT; Rpl7-r: CCGGATTTCTTTGCATTTCTTG

### Data Analysis

Results from color shift analysis, energy reserves assay, and transcriptional analysis data were analyzed by Student’s *t*-test and the Duncan’s test; data on body color reversibility were analyzed by Chi-square test; correlation analysis of energy reserves was performed using ANCOVA. All data were subjected to statistical analysis using the SPSS software (version 22; SPSS Inc., Chicago, IL, United States). The RAW images with 24-patch ColorChecker chart were analyzed using Adobe DNG Profile Editor (Version 1.0.0.46 beta, Adobe, CA, United States). All captured images were read and modified using Adobe Camera Raw (Version 7.1 beta, Adobe, CA, United States), and analyzed using Adobe Photoshop CS6 (Version 13.0 x64, Adobe, CA, United States).

## Results

### Values for the Color Channels in Color-Shifting Aphids

**FIGURE 1:**
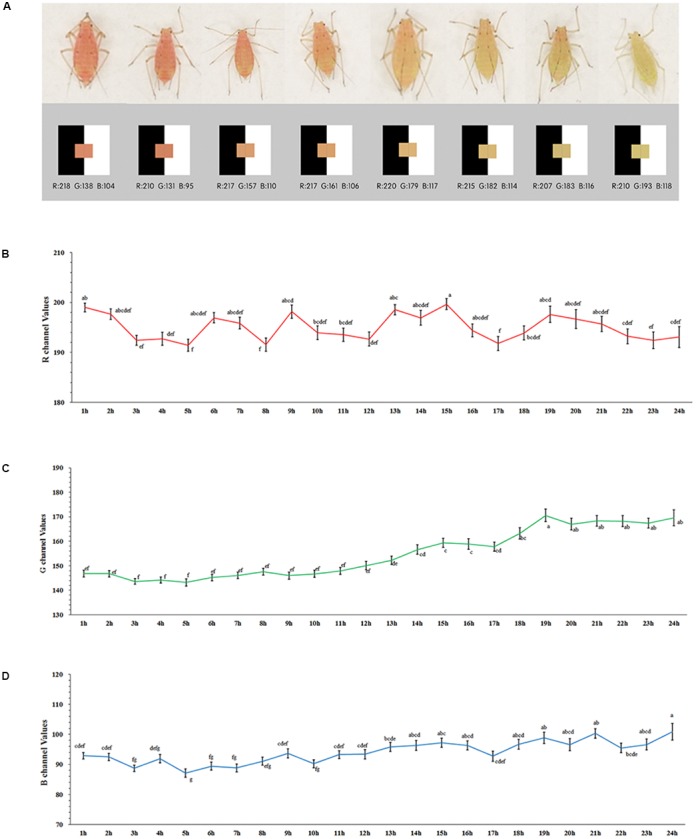
Values of RGB channel and body color of *Acyrthosiphon pisum* **(A)**, values of R **(B)**, G **(C)**, and B **(D)** dynamic change after 24 h of starvation. Different small letters above points indicate values that differ significantly among treatments (*P* < 0.05, Duncan test).

After the starvation treatment, the aphids showed variations in body color (Figure 1A). Although the values for the R channel varied greatly during the 24-h period (*F* = 2.105, *df* = 23, 1014, *P* = 0.002) (red), the changes were irregular (Figure 1B). The color channels moved to higher values during the red to pale red color shift as expressed by the values for both the G (*F* = 17.208, *df* = 23, 1014, *P* < 0.0001) (Figure 1C) and B channels (*F* = 6.758, *df* = 23, 1014, *P* = 0.002) (green and blue) (Figure 1D). The values of the G channel showed a marked increase, and the point in time at which the rise occurred was around 10 to 14 h after the starvation treatment, which plateaued at 19 h. The increasing trend of the B channel values was too weak to be identified (Figure 1D).

### Body Color Reversibility After Starvation

**FIGURE 2:**
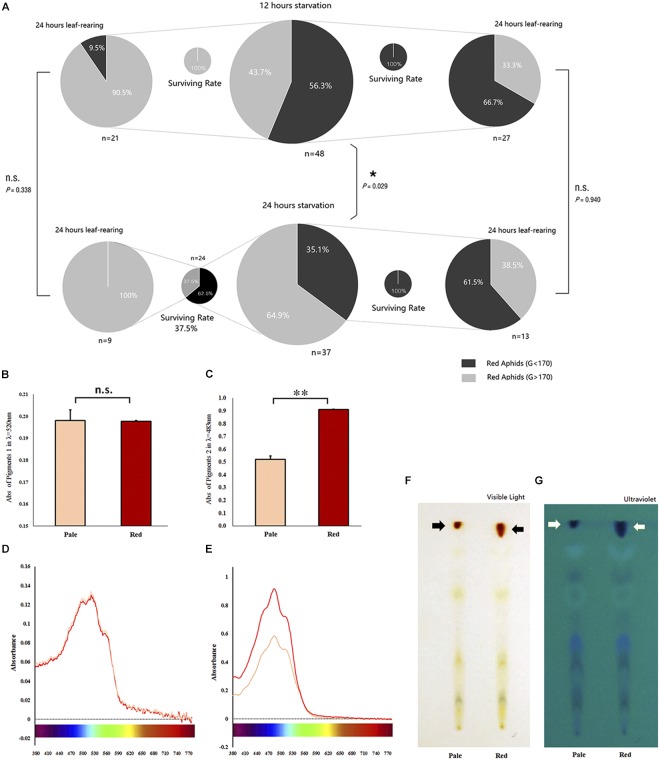
Survival of red *Acyrthosiphon pisum* in different body color under different conditions and the reddish pigments analysis. The proportion of experimental *A. pisum* in red show color shifting in 12 and 24 h after starvation; the red and pale were divided by G channel values, and the four smaller pies represent the surviving rates of each treatment **(A)**. Data represent statistics by Chi-square test (^∗^*P* < 0.05, ^∗∗^*P* < 0.01). Pigment contents of the red and pale *A. pisum* by absorbance and TLC. **(B,C)** The differences in absorbance in λ_max_ of each pigment; ^∗∗^ indicate significantly different at *P* < 0.001 (Student’s *t*-test). **(D,E)** UV–Vis waveforms in visible regions of two pigments. **(F,G)** TLC results under visible and ultraviolet lights.

Based on our pre-test, we selected the value of 170 as the shift point for the G channel. The proportion of body color shifted slightly (in pale red aphids, *G* > 170) and remained unchanged (in red aphids, *G* < 170) after 12- and 24-h starvation periods, which were separately but continuously monitored for color change. The 24-h starvation treatment stimulated a high proportion of aphids to shift color (χ^2^ = 4.773, *df* = 1, *P* = 0.029, Chi-square test); most of these color-shifted or slightly color-shifted aphids then turned to pale red (*G* > 170) in another 24 h after being reared on leaf disks in both treatments (χ^2^ = 0.918, *df* = 1, *P* = 0.338); about 1/3 of the aphids that had a red (*G* < 170) body color turned to pale red (*G* > 170) within another 24 h on leaf disks after the two treatments. The proportion of aphids that shifted to pale red was similar between the two treatments (χ^2^ = 0.006, *df* = 1, *P* = 0.940; Figure 2A).

The distributions of R, G, and B values of all the starved aphids before and after being reared on leaf disks for 24 h are shown in Supplementary Figure S3. The values for the G channel in both treatments were categorized into two groups with a cutting point of 170 (*G* value) after 24 h of rearing (values distribution, 12 h: before treatment, *P* = 0.036; after treatment, *P* < 0.0001; 24 h: before treatment, *P* < 0.0001; and after treatment, *P* = 0.012; Supplementary Figure S3). The values of the R channel maintained a normal distribution (12 h: K.S. test, before treatment, *P* = 0.200; after treatment, *P* = 0.184; 24 h: before treatment, *P* = 0.200; after treatment, *P* = 0.200). The values of the B channel showed similar trends (12 h: before treatment, *P* = 0.200; after treatment, *P* = 0.200; 24 h: before treatment, *P* = 0.200; and after treatment, *P* = 0.200; Supplementary Figure S3).

### Absorbance Patterns of Differently Colored *A. pisum* Pigments

Preliminary studies on the nature of pigments show some differences. The UV–Vis absorption spectral readings of the two crude extracts of the pigment are shown in Supplementary Figure S4. The pigment 1 solution in dark-red color showed two absorption bands: one at 240–300 nm (ultraviolet region) and another one at 340–590 nm (visible region, λ_max_ = 520 nm, Supplementary Figures S4A,B); the pigment 2 solution in orange-red color also had two absorption bands: one at 200–340 nm (ultraviolet region) and another one at 400–550 nm (visible region, λ_max_ = 483 nm, Supplementary Figures S4C,D). Both the absorption bans and the absorbance peak of pigment 1 solution were located in the visible region, close to the infrared region, which formed the yellow-green absorption band (550–600 nm); this gave this pigment mixture a yellow-green reflectance spectrum, and thus, the pigment 1 mixture appeared tomato red (Supplementary Figure S4B). The pigment 2 mixture, with its absorption band and absorbance peak located close to the ultraviolet region with little contribution toward the yellow-green absorption band, had a yellow-green reflectance spectrum; therefore, this mixture appeared as orange-red (Supplementary Figure S4D). Both pigment mixtures appeared to be oil-like in nature after they were concentrated; they were dried by vacuuming for 24 h (Supplementary Figure S4F).

### Detection of Pigments of the Two Different Colors in *A. pisum*

A comparative analysis of the two pigment solutions extracted from *A. pisum* revealed the amounts of red (G channel value around 160) and pale red (G channel value around 190) pigments to be similar in pigment 1 mixture (*t* = 0.056, *df* = 5, *P* = 0.957, Figure 2B), but significantly different in the pigment 2 mixture (*t* = −13.630, *df* = 3, *P* = 0.001, Figure 2C).

### Absorbance Potential of the Pigments

**FIGURE 3:**
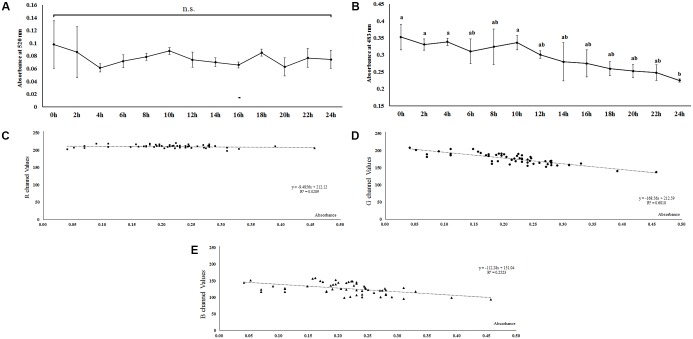
Dynamic changes of two reddish pigments mixtures extracted from *Acyrthosiphon pisum* after 24 h of starvation based on absorbance at λ_max_ of each pigment mixture **(A,B)**. Correlation between absorbance at λ_max_ (480 nm) of pigments 2 and R, G, B values **(C–E)** in *Acyrthosiphon pisum*. Pigments were extracted from the same aphids immediately after color collection. Different small letters above points indicate values that differ significantly among treatments (*P* < 0.05, Duncan test).

The contents of the crude extracts of the pigments, the pigment 1 and pigment 2, changed with the shifting of color in the aphid’s body (Figure 2D,E). The absorbance pattern of the pigment 1 mixture was irregular at λ_max_ = 520 nm and did not show any significant difference during the whole period of color shifting (*F* = 0.362, *df* = 12, 26, *P* = 0.966; Figure 3A); on the other hand, the absorbance pattern of the pigment 2 mixture showed a decreasing trend 12 h after starvation at λ_max_ = 483 nm (*F* = 1.737, *df* = 12, 26, *P* = 0.116; Figure 3B), which revealed that pigment 2 mixture might be responsible for the color shift.

### Correlation of Pigment Absorbance and the RGB Color Channels

The results of absorption (based on λ_max_ of pigment 2 solutions) and values for the RGB channels showed that there was no correlation between the pigment absorbance and values for the R channel (*r* = −0.170, *P* = 0.211, Kolmogorov–Smirnov test, Figure 3C). Values for both the G and B channels were negatively correlated to pigment absorbance; the values for the G channel showed a stronger correlation with absorbance than the values for the B channel (G channel, *r* = −0.826, *P* < 0.0001, Kolmogorov–Smirnov test, Figure 3D; B channel, *r* = −0.502, *P* < 0.0001, Kolmogorov–Smirnov test, Figure 3E).

### Thin-Layer Chromatography of Aphid Pigments

Thin-layer chromatography bands of extracts from *A. pisum* strains with the two different body colors (pale red and red, classified based on the value of G channel) were mostly similar in both their positions and quantities except for the red bands which were different; the extracts from red *A. pisum* had more red pigments (Figure 2F). Furthermore, TLC that was observed under ultraviolet light exhibited more bands than those observed in visible light; red pigments showed different contents between the two lanes (Figure 2G), but the two red pigments (pigment mixtures 1 and 2) could not be separated with this experimental protocol.

### Soluble Carbohydrate and Glycogen Assay

**FIGURE 4:**
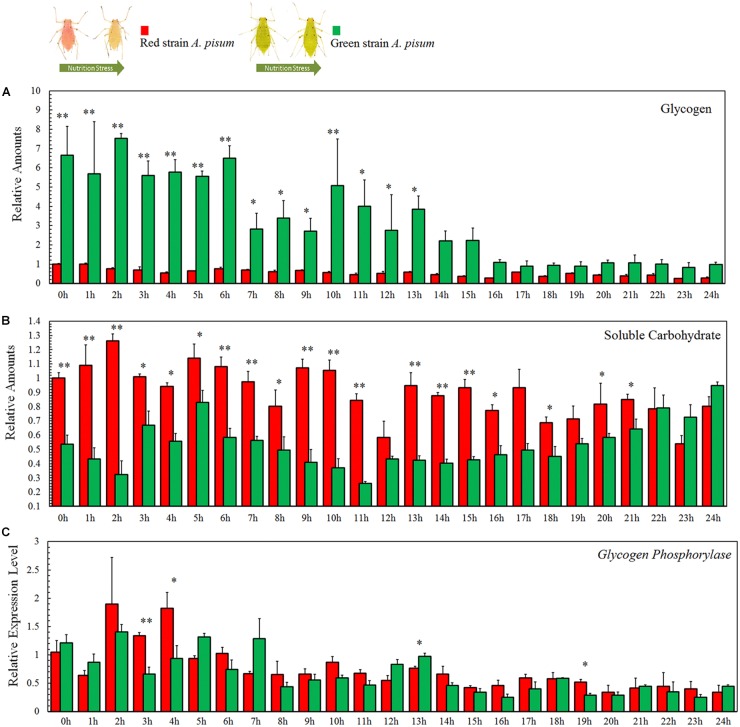
Soluble carbohydrate **(A)**, glycogen **(B)** reserves and relative transcription level of *glycogen phosphorylase* (*GP*), and **(C)** assay of *Acyrthosiphon pisum* in 24 h of starvation. ^∗^ and ^∗∗^ indicate that the means are significantly different at *P* < 0.05 and *P* < 0.001, respectively (Student’s *t*-test).

The change in carbohydrates is highly correlated to the degree of starvation treatment. The glycogen reserves in the earlier points in time were significantly different between the two strains of aphids (*P* < 0.001, tests of between-subjects effects, ANCOVA), while there were no significant differences in the later points in time. The green strain contained about sevenfold more glycogen than the red strain during the first 6 h, and this amount gradually decreased (Figure 3A). Although both curves displayed a decreasing trend, glycogen reserves in the green strain declined more rapidly than in the red strain. The differences in values recorded at each point in time were also analyzed by Student’s *t*-test and are marked in the diagrams (*P^∗^* < 0.05, *P*^∗∗^ < 0.001, Figure 4A).

The soluble carbohydrates displayed fluctuations in both the aphid strains. The tests of between-subjects effects (ANCOVA) showed strong correlations between time and experimental strains (*P* < 0.001); comparative experiments showed differences at many points in time between the two strains (Figure 4B). The dynamic changes in soluble carbohydrates in both strains went up and down during the first 12 h and then declined during the last 12 h (Figure 4B).

The differences in values at each time point were analyzed using Student’s *t*-test and are marked in corresponding diagrams (*P^∗^* < 0.05, *P*^∗∗^ < 0.001, Figure 4B).

### *Glycogen Phosphorylase* Transcriptional Expression

The metabolism of glycogen is not much different between the two aphid strains. Tests for the between-subjects’ effects (ANCOVA) on the transcription levels of *glycogen phosphorylase* showed no correlation between time and strains (*P* = 0.113). Expression levels of *glycogen phosphorylase* in both aphid strains showed a decreasing trend with no significant differences between the two strains (*P* = 0.113; Figure 4C). The differences of expression levels at each point in time were also analyzed by Student’s *t*-test and marked in the corresponding diagrams (*P^∗^* < 0.05, *P*^∗∗^ < 0.001, Figure 4C).

### Energy Reserves Assay Between the Red and Pale Red *A. pisum* (Red Strain)

**FIGURE 5:**
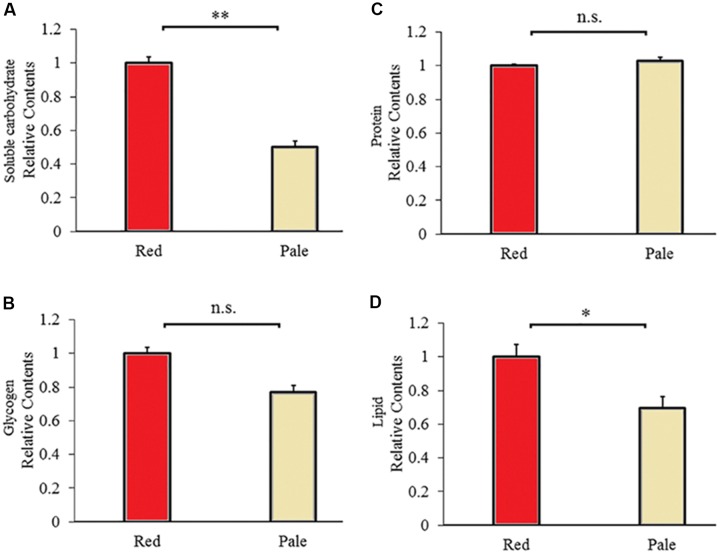
Carbohydrate **(A)**, glycogen **(B)**, protein **(C)**, and lipid **(D)** contents of the red and pale *Acyrthosiphon pisum* (based on G channel); ^∗^ and ^∗∗^ of **(D)** and **(A)** indicate significant different at *P* < 0.05 and *P* < 0.001, respectively (Student’s *t*-test).

There are differences in energy reserves contents between aphids before and after color fading. The red aphids contained significantly higher soluble carbohydrates (*t* = 9.592, *df* = 6, *P* < 0.0001, Figure 5A) and lipids (*t* = 2.711, *df* = 6, *P* = 0.035, Figure 5D) than the pale aphids. The contents of glycogen and proteins were not significantly different between the two strains of aphids (glycogen: *t* = 2.013, *df* = 10, *P* < 0.072; and protein: *t* = −1.284, df = 6, *P* = 0.228; Figure 5B,C).

## Discussion

Understanding shifts in the body color of *A. pisum* can help reveal the adaptation mechanisms to environmental threats in these aphids. We believe that in the red-colored strain of *A. pisum*, the change in body color is a response to nutritional stress, and thus, represents the aphid’s health status under such conditions. The reddish pigments are possibly used as backup energy reserves. The change in color was observed to occur in certain patterns and could be triggered under experimental conditions (starvation). Based on our results, we have concluded that the red *A. pisum* strain can remain red for about 10 h without food, after which it turns to a pale red color rapidly. Once an *A. pisum* initiates the color-shifting process, it cannot be stopped, regulated, or reversed even if the aphids are offered food. Between the two main pigment mixtures that were extracted in this study, the orange-red pigment mixture may be responsible for the color-shifting process and pigment degradation in *A. pisum*. All results from pigment analysis correlated with the color-shifting process. Carbohydrate and lipid contents correlated with color fading in the red *A. pisum* strain. The consumption patterns of energy reserves under starvation treatments between the two *A. pisum* strains were observed to be different; from this, we speculate that the reddish pigments may possibly be metabolized into carbohydrates for energy generation under nutrition stress, which may then lead to fading of color in the red *A. pisum* as well.

Based on the digital imagery analysis, we found out that the red *A. pisum* did not start color shifting during the first 10 h of starvation. Although it has been reported in previous studies that starvation stress can trigger color shift from red to pale red (Valmalette et al., 2012; Tabadkani et al., 2013), no detailed information has been provided. By using the digital imaging system, we were able to monitor the color change without interfering with them. The changes of aphids in color channel reflected the changes in the reflectance spectrum (Mancuso and Battiato, 2001; Vrhel et al., 2005). By analyzing the values for the RGB channels in the captured images, we found that the values for the R channel were always higher than those for the G and B channels. This might explain why the red *A. pisum* strain displays the red color in its body because there were no pigments that absorbed incoming red light in their body. Measurement values for the G and B channels showed that the aphids could reflect more green light than blue light (values for the G channel were higher than those for the B channel); the color of both bands of the reflected light (especially for G) was higher during the shift from red to pale red. We observed that the reflected red light during the shift from red to pale red did not show any change, while the green and blue lights increased, which made the reflected light whitish (Vrhel et al., 2005). Therefore, we assumed that some green-blue light absorbing pigments might have declined in their amount during the shift in body color in *A. pisum*, due to which they reflected more green-blue light and appeared to be pale red.

The red strain of *A. pisum* could remain red for about 10 h under no-diet conditions (Figure 1 and Supplementary Figure S2). We believe that *A. pisum* could not initiate the color-shifting process until the stimulus for it was sufficiently strong. The aphids that were still red (color shifting was not initiated) after treatment stayed reddish after they were re-transferred into better diet conditions (Supplementary Figures S2, S3), but the individuals whose body color had changed (even slightly) would eventually turn to the pale red color even after being re-transferred (Figure 2A and Supplementary Figure S3). It has been concluded that once the shift from red to pale red color in *A. pisum* is initiated, it cannot be stopped, until the process finishes.

The shift in body color is actually caused by a dynamic change in pigment composition and their contents. Pigments extraction and analysis showed that the ethyl alcohol-soluble pigment mixture with orange-red color might be responsible for the shift in color. The content of these pigments declined during the color-changing process. Earlier studies have reported that the content of some carotenoid pigments might be correlated to changes from red to pale red (Valmalette et al., 2012), but we could not identify each pigment in the pigment mixture so far. Based on earlier studies on the aphid pigments, these ethyl alcohol soluble pigments might be a type of aphins (Bowie et al., 1966; Horikawa et al., 2004, 2006). This pigments mixture showed a strong absorbance in the green band, a weak absorbance in the blue band, and almost no absorbance in the red band. Our absorbance analyses showed that the change in absorbance correlated with the fluctuation in the values for the G and B channels as well, indicating that the green-blue-absorption pigments degraded during the process of color shift. The TLC experiments showed similar results, where the red pigments in the pale red *A. pisum* strain were present in a relatively small amount. From this observation, we suggest that these reddish pigments are most likely degraded and metabolized for some other use. The details of such a process need to be further investigated.

**FIGURE 6:**
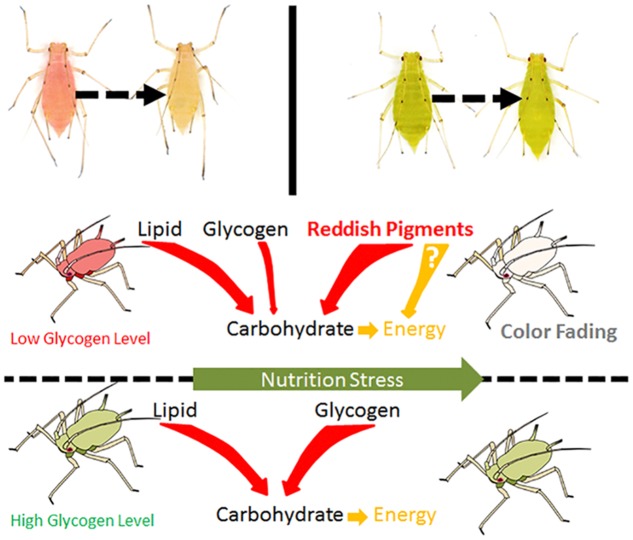
The hypothesis of energy consuming strategies differences between red and green strain aphids *Acyrthosiphon pisum* under nutrition stress.

Considering that nutrition stress is a key trigger for the fading of color, pigment degradation and conversion are probably related to nutritional physiology. Previous studies have shown that energy reserves (soluble carbohydrate and lipid contents) are different in the red and pale red *A. pisum* strains (Tabadkani et al., 2013). After monitoring the changes in the energy reserves during color shift from red to pale red, we found that there is a connection between shift in body color and energy reserves. The results showed that changes in both lipid and carbohydrate contents have similar trends as those in color shifts. As soluble carbohydrates could be formed from glycogen and lipids, or even from some other precursors, the soluble carbohydrate content did not drop dramatically and was maintained at a certain level (Figure 4 and Supplementary Figure S5). The energy reserves in red and pale red *A. pisum* strains were similar to those reported in literature (Tabadkani et al., 2013). The functions of these specific pigments in the red strain of *A. pisum* are unclear, so we conducted a comparative analysis of energy reserves with the green strain of *A. pisum*, which is genomically quite identical but has a different body color. Based on these comparative experiments, a significantly lower glycogen reserve was detected in the red strain than in the green strain; on the contrary, the carbohydrate level was slightly higher in the red strain. Based on the carbohydrate contents and absorbance patterns under starvation treatment, we assumed that there might be a way of carbohydrate refueling besides metabolization of glycogen in the red strain of *A. pisum*; these reddish pigments are the most likely candidates for being a precursor of such a process. The conversion of reddish pigments to carbohydrates may lead to the fading of the body color. It is also possible that the reddish pigments provide energy in some other pathway; more studies are needed to better understand this energy support system (Figure 6).

## Conclusion

The shift in body color from red to pale red in the red *A. pisum* strain depicts an adaptation mechanism to environmental changes; this color shift also reveals that *A. pisum* strains of different body colors exhibit diverse strategies of storing and using energy reserves. The connection between body color and *A. pisum* health status is a representation of the interactions between aphids and their environmental conditions from a new perspective. The shift in body color is actually a result of pigment degradation and even their consumption as energy reserves. Future studies should include the extraction and identification of key pigments, understanding of their molecular structures, related downstream metabolic pathways, especially the downstream metabolism, and conversion to reddish pigments.

## Author Contributions

X-XW, YZ, and T-XL designed research and wrote the manuscript. X-XW performed the research. J-YZ, Z-SC, and Z-JF provided assistance. YZ and X-XW analyzed the data.

## Conflict of Interest Statement

The authors declare that the research was conducted in the absence of any commercial or financial relationships that could be construed as a potential conflict of interest.

## Footnotes

1 https://enzyme.expasy.org/EC/2.4.1.1

2 http://www.ncbi.nlm.nih.gov/tools/primer-blast/index.cgi?LINK_LOC=BlastHome
