# Supplementary material for: Starvation Stress Causes Body Color Change and Pigment Degradation in Acyrthosiphon pisum
Source: Front Physiol. 2019 Mar 5;10:197. doi: 10.3389/fphys.2019.00197 (PMC6412094; doi:10.3389/fphys.2019.00197)
Supplement: Supplementary file 1 [file Presentation_1.pdf]

Supplementary material

Fig.S1. Technical protocol in color collection (A) and pigments extraction (B) from *Acyrthosiphon pisum.*

Fig.S2. Body color shifting (based on G channel) of five *Acyrthosiphon pisum* individuals in 24 hours starvation. Color collection protocol: Twelve hours old wingless fourth instar nymphs of the red A. pisum were used in this color-shifting test. The nymphs were individually placed into a 24-well tissue culture plate (transparent plastic; 1 individual per well) for starvation treatment. The color-shifting images of the aphids were individually captured at hourly intervals for 24 h. The values of RGB channels of each aphid were read by Adobe Photoshop CS6 software.

Fig.S3. *Acyrthosiphon pisum* distribution based on RGB channel values of body-color in 12 (A, B) and 24(C, D and E) hours starvation. B and E represent the distribution based on color of aphids being re-transferred into better diet condition for 24 hours. D represents the survivors' distribution based on color of aphids treated by 24 hours starvation.

Fig. S4. Two reddish pigments mixture extracted from *Acyrthosiphon pisum* and absorbance analysis, UV-Vis waveforms were generated by Nanodrop 2000c spectro photometer. pigments mixture 1 (A UV-Vis-IR region, B Visible region) and pigments mixture 2 (C UV-Vis-IR region, D Visible region), appearance of crude extraction of two pigments mixture shows in E and F.

Fig. S5. Lipid (A) and Protein (B) reserves level assay of *Acyrthosiphon pisum* in 24 hours of starvation. * and ** indicate that the means are significantly different at $P<0.05$ and $P<0.01$, respectively (Student's *t*-test).

Fig.S1

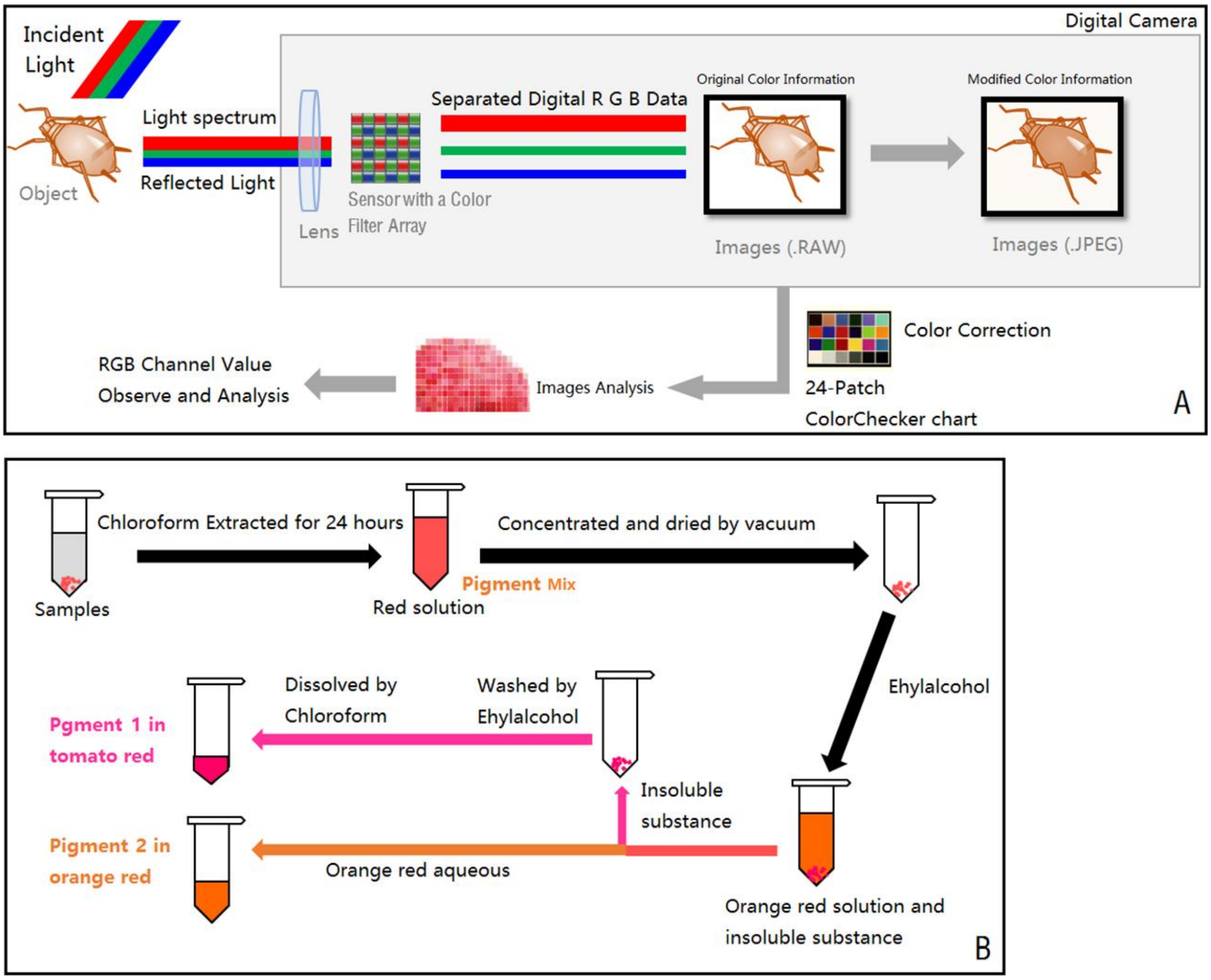

Digital Camera
Incident Light
Light spectrum
Reflected Light
Object
Lens
Sensor with a Color Filter Array
Separated Digital R G B Data
Original Color Information
Images (.RAW)
Modified Color Information
Images (.JPEG)
Color Correction
24-Patch ColorChecker chart
Images Analysis
RGB Channel Value Observe and Analysis
A
Chloroform Extracted for 24 hours
Concentrated and dried by vacuum
Samples
Red solution
Pigment Mix
Ehylalcohol
Dissolved by Chloroform
Washed by Ehylalcohol
Pgment 1 in tomato red
Insoluble substance
Pigment 2 in orange red
Orange red aqueous
Orange red solution and insoluble substance
B

Fig.S2

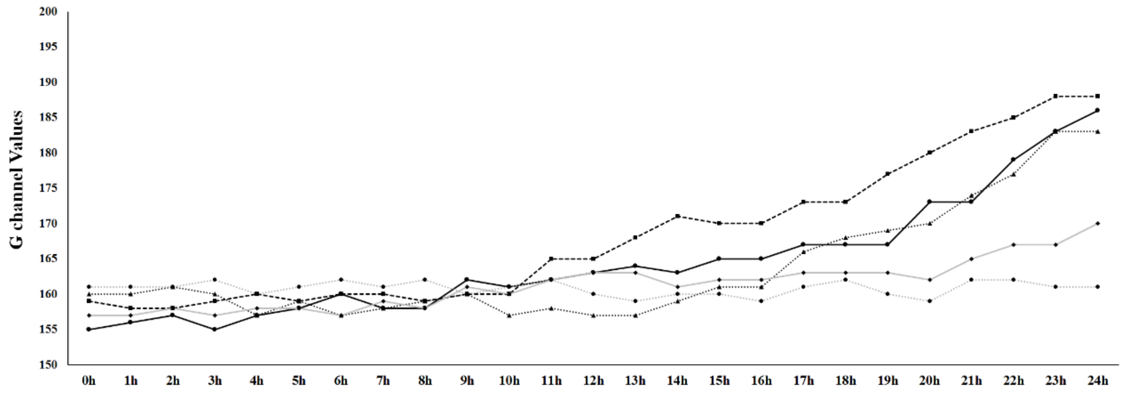

G channel Values
200
195
190
185
180
175
170
165
160
155
150
0h
1h
2h
3h
4h
5h
6h
7h
8h
9h
10h
11h
12h
13h
14h
15h
16h
17h
18h
19h
20h
21h
22h
23h
24h

Fig.S3

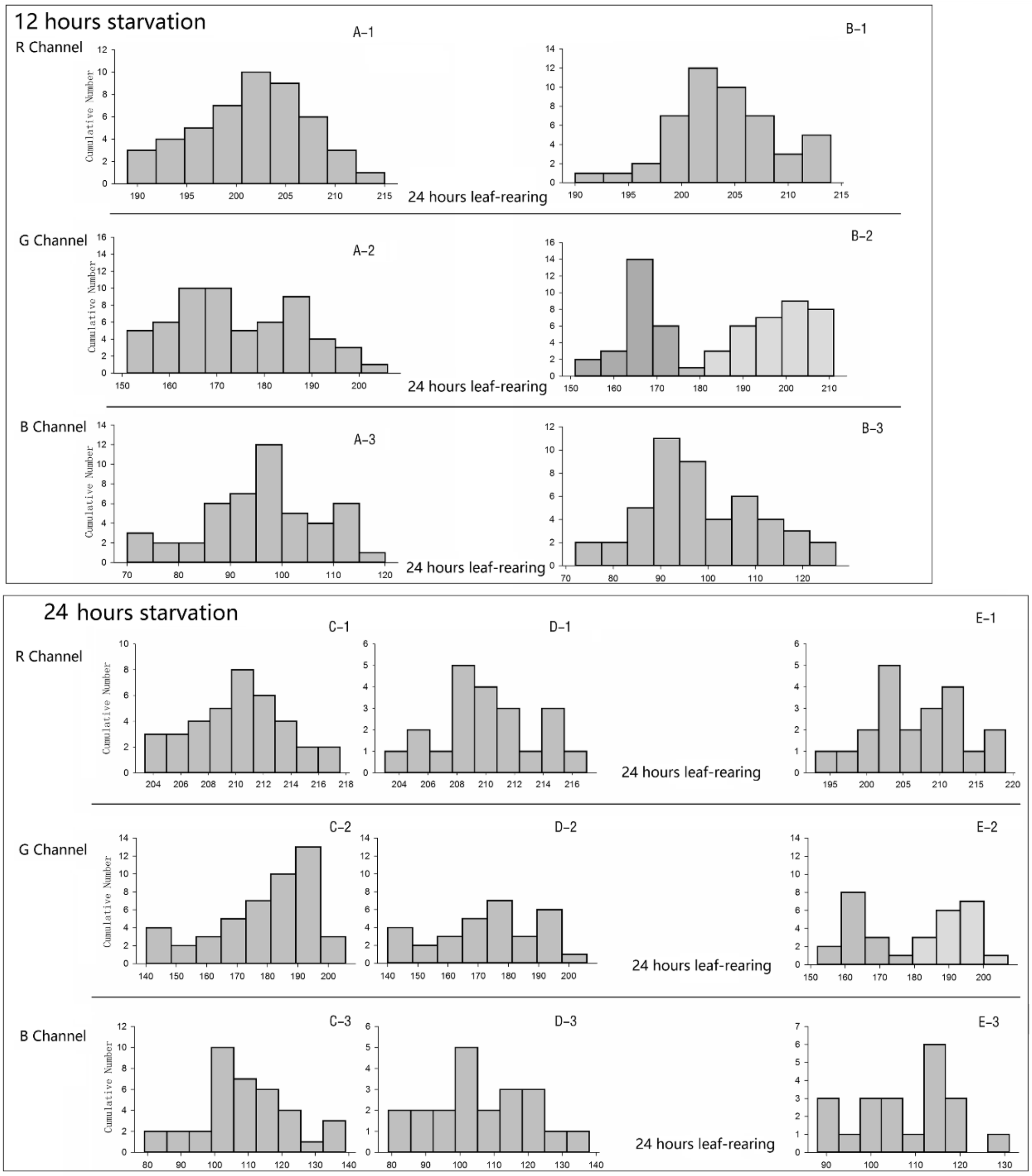
12 hours starvation
R Channel
G Channel
B Channel
A–1
B–1
A–2
B–2
A–3
B–3
Cumulative Number
24 hours leaf-rearing
24 hours starvation
C–1
D–1
E–1
C–2
D–2
E–2
C–3
D–3
E–3

Fig.S4

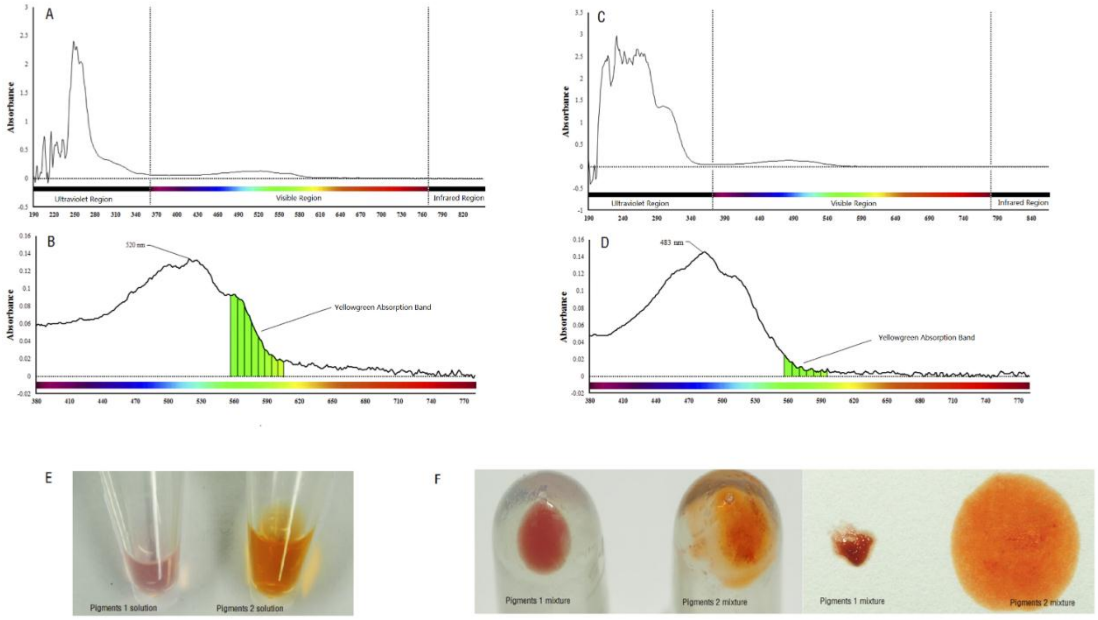
A
B
C
D
E
F
Absorbance
Ultraviolet Region
Visible Region
Infrared Region
520 nm
483 nm
Yellowgreen Absorption Band
Pigments 1 solution
Pigments 2 solution
Pigments 1 mixture
Pigments 2 mixture
Pigments 1 mixture
Pigments 2 mixture

Fig.S5

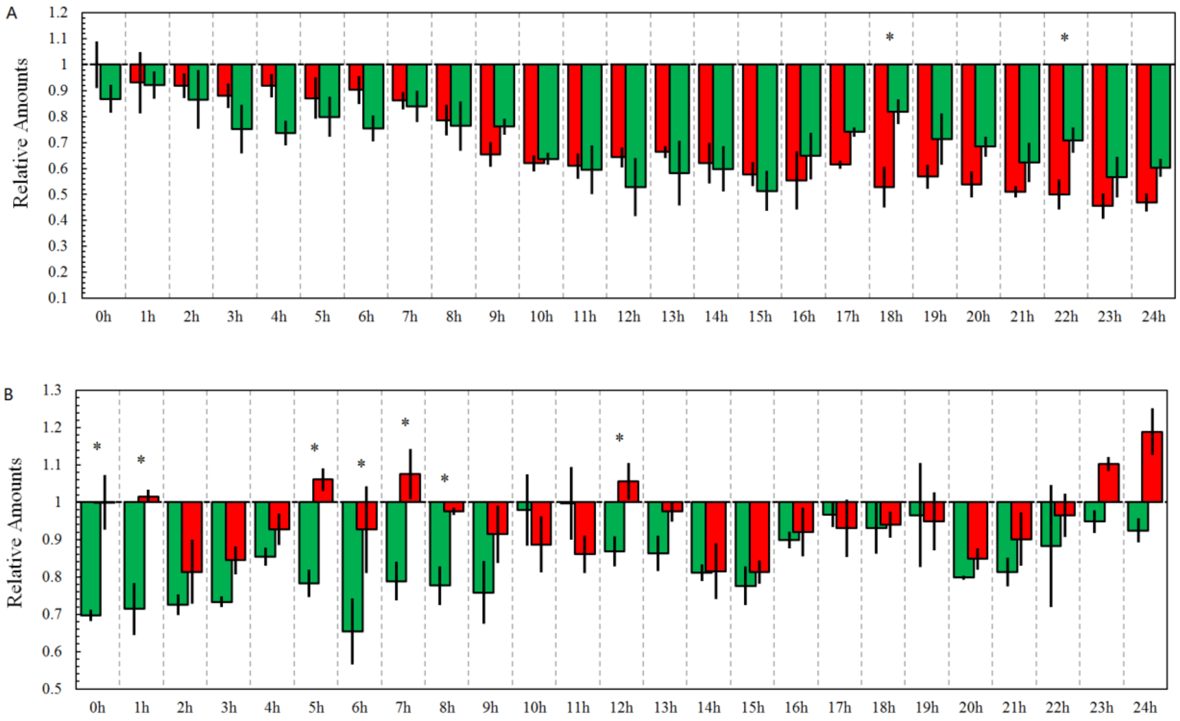
A
Relative Amounts
0h 1h 2h 3h 4h 5h 6h 7h 8h 9h 10h 11h 12h 13h 14h 15h 16h 17h 18h 19h 20h 21h 22h 23h 24h
B
Relative Amounts
0h 1h 2h 3h 4h 5h 6h 7h 8h 9h 10h 11h 12h 13h 14h 15h 16h 17h 18h 19h 20h 21h 22h 23h 24h
